# Focus on Non-osteoporotic Post-menopausal Women with Asymptomatic Primary Hyperparathyroidism: A Monocentric Series

**DOI:** 10.2174/0118715303288678240424074110

**Published:** 2024-05-07

**Authors:** Elena Castellano, Roberto Attanasio, Alberto Boriano, Laura Gianotti, Giorgio Borretta

**Affiliations:** 1 Department of Endocrinology, Diabetes and Metabolism, Santa Croce and Carle Hospital, Cuneo, Italy;; 2 Scientific Committee, Associazione Medici Endocrinologi, Milano, Italy;; 3 Department of Medical Physics, Santa Croce and Carle Hospital, Cuneo, Italy

**Keywords:** Postmenopausal women, primary hyperparathyroidism, osteoporosis, asymptomatic PHPT (aPHPT), fracture risk, FRAX

## Abstract

**Objective:**

Primary Hyperparathyroidism (PHPT) is a common disease, frequently diagnosed in post-menopausal women, among whom Osteoporosis (OP) is a common finding. To date, no study has specifically evaluated the asymptomatic PHPT (aPHPT) patients without OP, in particular post-menopausal women who are exposed to an increased risk of developing OP.

**Materials and Methods:**

This study involved a retrospective cross-sectional evaluation. From our database of 500 consecutive patients diagnosed with PHPT, 178 post-menopausal aPHPT were retrieved.

**Results:**

The clinical, biochemical, and imaging data of the 85 patients without OP were not different from those of the 93 with OP, except for bone alkaline phosphatase (significantly higher in the latter group). Among these 85 patients without OP, the 45 patients meeting surgical criteria for parathyroidectomy had significantly higher values of serum PTH (240 *vs*. 99 ng/L, *p* =0.03) and calcium (total, 11.2 ± 0.7 *vs*. 10.6 ± 0.4 mg/dL, *p* <0.001; ionized, 1.45 ± 0.12 *vs*. 1.36 ± 0.8 mmol/L, *p* =0.044) and lower values of serum phosphate (2.57 ± 0.7 *vs*. 2.94 ± 0.5 mg/dL, *p* =0.009) and eGFR (68.5 ± 23.8 *vs* 80.8 ± 14.4 mL/min/1.73 m^2^, *p* =0.006) than the 40 aPHPT patients not meeting surgical criteria, without any difference in densitometric data and calculated fracture risk.

**Conclusion:**

In our series, post-menopausal aPHPT patients without OP accounted for almost a sixth of the whole PHPT series. About half of these patients did not meet surgical criteria, but their T scores and 10-year fracture risk calculated by FRAX were not significantly different from post-menopausal aPHPT without OP meeting surgical criteria.

## 1 INTRODUCTION

The clinical and epidemiological presentation of sporadic Primary Hyperparathyroidism (PHPT) has changed profoundly over the last few decades, shifting to a largely asymptomatic disease (aPHPT).

PHPT is more frequently diagnosed in post-menopausal women [1, 2], among whom Osteoporosis (OP) is a common finding [3, 4]. OP is the criterion for surgery most frequently met in aPHPT, according to the international guidelines for aPHPT management [5].

To date, no study has specifically evaluated aPHPT patients without OP, in particular post-menopausal women, who are by definition exposed to an increased risk of developing OP [6, 7].

We have, thus, evaluated the prevalence of aPHPT without OP in a large unselected consecutive series of PHPT patients, focusing on the clinical features of post-menopausal aPHPT patients. Furthermore, among these patients, we have compared the clinical profile of those meeting or not surgical indications according to the latest international guidelines.

## 2 MATERIALS AND METHODS

### 2.1 Design

We conducted a retrospective survey on the medical records of all patients diagnosed with PHPT who presented at our department between January 1997 and December 2021.

The study was conducted in accordance with the Declaration of Helsinki. Patients gave informed consent for these investigations (as part of their normal medical care both at diagnosis and follow-up). The study was approved by the institutional review board and the ethics committee of our institution (ID ENDO38).

### 2.2 Patients

The patients included in our study were referred by general practitioners, subspecialty clinics, and primary care clinics.

Patients of both sexes were included; gender data were collected and entered into our database as dichotomous variables, and no limitations or challenges were encountered in our series.

The diagnosis of PHPT has been established by the presence of hypercalcemia and concomitant inappropriately high levels of serum PTH on at least two separate occasions. We excluded patients diagnosed with multiple endocrine neoplasia, hyperparathyroidism-jaw tumor syndrome, familial hypocalciuric hypercalcemia, parathyroid carcinoma, and normocalcemic PHPT. None of the included patients had been taking calcium or vitamin D supplements, estrogens or testosterone or selective estrogen receptor modulators, or bone-active medications for at least 6 months.

In agreement with the study of Bilezikian *et al*. [5], patients were classified as having aPHPT on the basis of a lack of radiological signs of bone involvement, nephrolithiasis, and symptoms of hypercalcemia. All patients had routinely undergone dual X-ray Absorptiometry (DXA) and a radiographic evaluation of the skull and hands to check for signs of excess PTH effects on the bones, such as subperiosteal resorption in the fingers, salt and pepper mottling of the skull, or brown tumors (osteitis fibrosa cystica).

The fracture risk assessment (FRAX) tool [8], calibrated for the Italian population, was used to calculate the 10-year risk of Hip Fracture (HF) and major Osteoporotic Fracture (OF) in the subgroup without OP.

As for kidney involvement, patients were classified as symptomatic either if they had a recorded positive history of renal stones or if renal stones or calcinosis had been diagnosed by routinely performed ultrasound in either asymptomatic or symptomatic patients.

The aPHPT patients not meeting the surgical criteria set out by the latest international guidelines [5] and retrospectively applied to all patients were considered “mild asymptomatic” patients [9].

From our database of consecutive patients diagnosed with PHPT [10, 11], for the present study, we extracted the data of aPHPT patients.

### 2.3 Methods

All blood samples were collected after overnight fasting and rest. As reported in previous studies of our group [10, 11], serum total Ca, P, and creatinine as well as Urinary Ca (UCa) were analyzed by a standard auto-analyzer using colorimetric and enzymatic methods, whereas ionized serum Ca was analyzed by a specific probe after correction for pH.

The estimated Glomerular Filtration Rate (eGFR) was calculated using the CKD-EPI formula [12].

Serum intact PTH concentrations were measured up to 2012 using a two-site immunochemiluminometric assay and then using a new second-generation immunochemiluminometric assay (Cobas e411, Roche Diagnostics).

Serum 25-hydroxy-vitamin D (25OHD) levels were measured by a radioimmunoassay (DIAsource 25OHVit. D3-Ria-CT Kit, DIAsource Immuno Assays S.A., Nivelles, Belgium).

The levels of bone-specific Alkaline Phosphatase isoenzyme activity (bALP) and osteocalcin were measured by chemiluminescent evaluation (CLIA, DiaSorin LIAISON XL).

Bone Mineral Density (BMD) was measured at the lumbar spine (L1-L4), proximal femur, and distal third of the non-dominant radius using the same instrument (DXA QDR-4500, Hologic, Bedford, MA) throughout the study period. Minor upgrades to the BMD instrument did not significantly affect the results. According to the WHO criteria, in post-menopausal women, osteoporosis is defined as a T-score below 2.5 SD [13].

All patients underwent standard renal ultrasound using a 2- to 5-MHz-wide band convex transducer. For a definitive diagnosis of stones, radiologists looked for hyperechogenic spots more than 2 mm in diameter with a multiplanar evaluation of specific signs.

### 2.4 Statistical Analysis

Variables were preliminarily tested for normal distribution with the Shapiro-Wilk W test and data have been expressed as mean ± Standard Deviation (SD) or median and Interquartile Range (IQR) when normally distributed or not normally distributed, respectively.

Continuous variables with non-normal and normal distribution were analyzed by Mann-Whitney U test and t-test for unpaired samples, respectively, as appropriate. Differences in categorical variables were analyzed by χ^2^ or Fisher’s test, as appropriate. The level of statistical significance was set at *p* ≤0.05. All calculations were performed using SPSS (IBM SPSS Statistics - version 21).

## 3 RESULTS

From a database of 500 consecutive patients diagnosed with PHPT, for the present study, we focused on the 85 post-menopausal aPHPT patients without OP at any site, representing the 17% (85/500) of the whole PHPT series, the 26.2% (85/324) of post-menopausal PHPT, and the 47.8% (85/178) of post-menopausal aPHPT.

Fig. (**1**) shows the distribution of post-menopausal aPHPT with and without OP in different age groups (*p* = 0.018). Table **1** compares demographic, clinical, and biochemical parameters between the 85 post-menopausal aPHPT patients without OP and the 93 post-menopausal aPHPT with OP at any site.

Except for densitometric parameters, impaired by definition being the dividing parameters, only bALP levels resulted significantly higher in the OP subgroup.

The 85 post-menopausal aPHPT patients without OP were then subdivided according to their fulfillment of surgical criteria (Table **2**); PTH and calcium (serum total and ionized, and UCa) levels were significantly higher and serum phosphate and eGFR were significantly lower in the 45 aPHPT patients meeting surgical criteria than in the 40 patients with mild PHPT. No differences were found either in the mean T score at any site or in the FRAX-calculated 10-year fracture risk of HF and OF, and the rate of pre-surgical localization was similar between the two subgroups. The rate of osteopenia at the radial level, but not at the spine or femur, was significantly higher in the aPHPT meeting surgical criteria subgroup.

Finally, the 85 post-menopausal aPHPT patients without OP were evaluated according to age. Patients over 65 years, so-called “older adults”, despite similar PTH and serum Ca levels, had significantly lower UCa (183.4 (250) *vs*. 277.7 (288) mg/24 h, *p* = 0.02) and eGFR levels (68.8 ± 19.6 *vs*. 80.1 ± 20.1 mL/min/1.73 m^2^, *p* = 0.013) than the youngers. BMD at the distal radius was significantly lower in older adults than in younger patients (T score -1.56 ± 0.83 *vs*.-0.919 ± 0.95, *p* = 0.047), without any differences at the other sites.

## 4 DISCUSSION

In our study, post-menopausal women without OP constituted the largest group among aPHPT patients without OP (85/125 = 68%), accounting for almost a sixth of the whole PHPT series. Moreover, the prevalence of aPHPT without OP decreases with increasing age at diagnosis of post-menopausal aPHPT.

In almost half of cases (40/85 = 47%), post-menopausal aPHPT without OP did not meet the surgical criteria recommended by the latest guidelines and represented the large majority of mild aPHPT patients.

Among post-menopausal aPHPT women without OP, those meeting surgical criteria had a more severe biochemical disease expression. The prevalence of radial osteopenia was higher in the aPHPT meeting surgical criteria subgroup than in mild PHPT patients. However, mean T scores and 10-year fracture risk were not different between post-menopausal aPHPT patients without OP meeting the surgical criteria or not. Finally, BMD at the distal radius was significantly lower in older adults than in younger post-menopausal aPHPT patients.

PHPT is common in post-menopausal women, with an asymptomatic clinical presentation in most cases [3, 4, 11]. OP is a common finding in post-menopausal PHPT [4, 11].

Many factors could explain these findings. The post-menopausal estrogen drop seems to play a role in parathyroid tumorigenesis [14, 15], thus contributing to the increased prevalence of PHPT in post-menopausal women. Moreover, in Western countries, the widespread biochemical screening, decreased prevalence of vitamin D deficiency, and the assessment of PTH levels as part of the evaluation for low bone mass, allow the recognition of pauci-asymptomatic forms of PHPT, particularly in this population [16, 17].

Moreover, in our series, almost half of post-menopausal aPHPT patients without OP had a mild disease.

Recent observational studies have reported that in real practice, PTX is underutilized as a definitive treatment for PHPT [4, 18, 19]. In particular, aPHPT patients without OP [4] and mild aPHPT are more frequently managed with a non-operative approach [18]. However, increasing evidence points to PTX-induced reduction of fracture risk across different skeletal sites [19] in all aPHPT patients [20-23] regardless of OP [24].

Prospective controlled studies [25-29] have shown successful PTX to achieve an improvement in BMD even in patients with mild aPHPT, differently from what has been observed in the surveillance group. However, the only available long-term study did not report any detectable difference in fracture risk among patients with mild aPHPT randomly assigned to PTX or observation [29].

In our series, T scores were not different between post-menopausal aPHPT patients without OP subdivided according to fulfillment of surgical indication.

Several clinical factors are associated with fracture risk. In the last decades, FRAX has been extensively used for the prediction of fracture risk in the general population, but its use in PHPT patients has been criticized [30]. Recent studies have suggested, however, that FRAX may be reliable also in PHPT patients, provided that calibrated versions for different countries are employed [21].

Not with standing its limitations, the FRAX score provides a comprehensive risk assessment greater than the DXA measurement alone [21]. With the use of FRAX, we found the 10-year fracture risks of HF and OF to not be different between post-menopausal aPHPT without OP meeting or not the surgical criteria. In addition to a similar fracture risk profile, the pre-surgical localization rate was not different between post-menopausal aPHPT without OP meeting or not the surgical criteria.

The prevention of fragility fracture is a public health priority worldwide [6] and the guidelines for the prevention and management of osteoporosis [7, 31] underline that prevention is primarily based on the correction of all modifiable risk factors. Post-menopausal status is by definition a non-modifiable risk factor for OP [7, 31], because estrogen drop causes accelerated bone loss [29]. PHPT is a recognized cause of secondary OP [7, 31] and fracture rates in PHPT patients are estimated to be 1.5-fold greater than in the general population [20, 32, 33].

In our series, as in the general population [22], OP has become more frequent with increasing age. The probability of not having OP in post-menopausal women is higher in younger patients, where usually the comorbidity burden and the risk of perioperative complications associated with PTX are lower [34].

Longer life expectancy may thus justify actions that are aimed at preventing OP development and reducing the fracture risk [7, 35].

The significantly more impaired T score at the radial level found in older post-menopausal PHPT patients without OP compared to the younger patients is specific to PHPT involvement [1, 2, 13]. It might be associated with a longer disease duration [35], and thus indirectly point to a delayed diagnosis of PHPT. For this reason, early recognition of the disease after menopause could extend the likelihood of identifying aPHPT before the development of OP, and thus allow counseling to remove a modifiable risk factor of secondary OP.

However, to date, international guidelines for PHPT management [5] do not support early screening for aPHPT after menopause.

Our study has several limitations. Despite the large cohort studied, this was a retrospective, single-institution study, which may have been affected by selection bias. The findings cannot, therefore, be generalized indiscriminately to PHPT patients in other countries and ethnic groups. However, unlike previous studies, this study has evaluated a consecutive series of patients, and thus may have better reflected real-life clinical practice. Moreover, even though we have excluded patients taking bone-active medications in the last six months, we cannot rule out a persisting bone effect of a more remote therapy with these drugs. The population and the phenotypic descriptor data for this study have been acquired over a prolonged period of time, raising the hypothetical possibility that background variables may have changed over this time. However, as previously reported by our group, the clinical presentation of PHPT has been found to be stable over 20 years in our series [36].

Finally, we acknowledge that we did not use the gold standard ‘liquid chromatography aligned to mass spectrometry’ to assess 25OHD levels and also that our RIA may have slightly overestimated 25OHD levels (in line with several RIAs). However, all measurements were performed in the same laboratory, thereby ensuring a good quality of data.

## CONCLUSION

In conclusion, in our study, post-menopausal aPHPT patients without OP accounted for almost a sixth of the whole PHPT series. About half of these patients had mild disease, but their T scores and 10-year fracture risk calculated by FRAX were not significantly different from post-menopausal aPHPT patients without OP meeting the surgical criteria.

Early recognition of aPHPT after menopause could extend the likelihood of identifying patients without OP and acting as a modifiable risk factor for secondary OP. Longer-term prospective studies on large patient series are needed to test this hypothesis.

## Figures and Tables

**Fig. (1):**
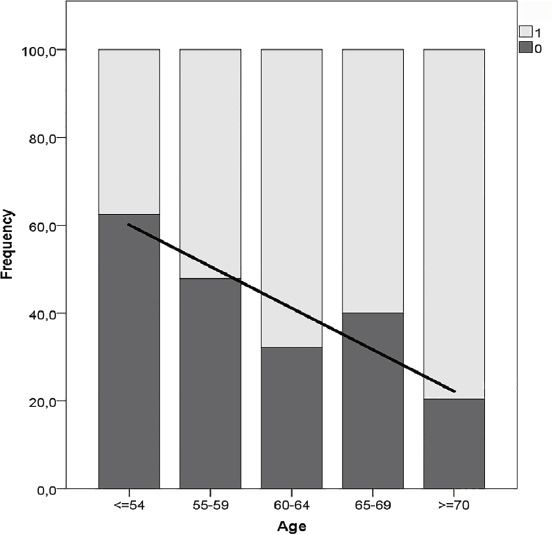
Distribution of post-menopausal aPHPT women with and without osteoporosis (OP) in different age groups (year range in x-axis). Black bars (0) = no OP at any site. Empty bars (1) = presence of OP.

**Table 1:** Comparison between the postmenopausal aPHPT patients without and with OP at any site.

**-**	**Without OP (n = 85)**	**With OP (n = 93)**	p*
**Age** (years) **Older adults** n (%)	65.8 ± 9.5 41 (48.2%)	67.7 ± 8.3 49 (52.7%)	0.164 0.329
**PTH** (ng/L)	173 [60.75]	169 [128.1]	0.909
**Total serum Calcium** (mg/dL)	10.9 ± 0.7	11 ± 0.7	0.287
**Ionized Calcium** (mmol/L)	1.41 ± 0.1	1.42 ± 0.1	0.402
**Serum Phosphate** (mg/dL)	2.75 ± 0.83	2.8 ± 0.56	0.537
**Urinary Calcium** (mg/24h)	235 ± 152	248.8 ± 153	0.634
**Urinary Phosphate** (mg/24h)	599 ± 326	584 ± 358	0.853
**eGFR** (mL/min/1.73 m^2^)	74.6 ± 20.5	78.2 ± 21.2	0.150
**BMI** (kg/m^2^)	26.4 ± 5.9	25.1 ± 4.5	0.133
**Total Alkaline Phosphatase** (U/L)	110.9 ± 59.8	110.3 ± 51.7	0.951
**Bone-specific alkaline phosphatase** (U/L)	18 ± 9.8	24.1 ± 16	**0.018**
**Osteocalcin** (ng/mL)	37.7 ±7	44.5 ±5.2	0.294
**25OHD** (μg/L)	27.7 ± 15.8	28.2 ± 18.7	0.891
**Lumbar T score**	-1.36 ± 0.95	-3.1 ± 0.97	**
**Distal third radius T score**	-1.2 ± 0.95	-3.2 ± 1.1	**
**Femoral T score**	-1.3 ± 0.7	-2.4 ± 0.9	**
**Lumbar Z score**	0.32 ± 1.1	-1.2 ± 1.3	**
**Distal third radius Z score**	0.22 ± 0.8	-1.3 ± 0.9	**
**Femoral Z score**	0.1 ± 0.7	-0.7 ± 0.9	**
**aPHPT meeting surgical criteria mild aPHPT**	52.9% 47.1%	100% /	-
**Positive imaging (US or SestaMIBI)**	76.4%	83.7%	0.212

**Abbreviations:** aPHPT: asymptomatic primary hyperparathyroidism; OP: osteoporosis; §: over 65 years; US: ultrasonography. **Note:** Data are expressed as mean ± SD when normally distributed, median and [IQR] when not normally distributed and as absolute number and percentage when categorical. *Bold points to statistically significant difference. **Data reported only for description because this is the dividing variable for the two groups.

**Table 2:** Comparison between post-menopausal aPHPT patients without OP meeting or not surgical criteria.

**-**	**Meeting Surgical Criteria (n = 45)**	**Not Meeting Surgical Criteria (n = 40)**	p*
**Age** (years)	65.4 ± 9.1	66.3 ± 10.2	0.710
**PTH** (ng/L)	240 [126]	99 [60]	**0.03**
**Total serum Calcium** (mg/dL)	11.2 ± 0.7	10.6 ± 0.4	<0.001**
**Ionized Calcium** (mmol/L)	1.45 ± 0.12	1.36 ± 0.8	**0.044**
**Serum Phosphate** (mg/dL)	2.57 ± 0.7	2.94 ± 0.5	**0.009**
**Urinary Calcium** (mg/24h)	276 ±163	185 ±123	0.025**
**Urinary Phosphate** (mg/24h)	604 ± 225	595 ± 238	0.925
**eGFR** (mL/min/1.73 m^2^)	68.5 ± 23.8	80.8 ± 14.4	0.006**
**BMI** (kg/m^2^)	25.7 ± 5.7	27.2 ± 6.3	0.323
**Total Alkaline Phosphatase** (U/L)	114.7 ± 60.8	106.6 ± 59.6	0.609
**Bone-specific alkaline phosphatase** (mg/L)	18.6 ± 10.6	17.4 ± 9.2	0.667
**Osteocalcin** (ng/mL)	47.8 ± 65.4	26.3 ± 10.7	0.06
**25OHD** (μg/L)	32.2 ± 18.3	24.5 ± 13.1	0.129
**Lumbar T score**	-1.2 ± 1.1	-1.5 ± 0.8	0.345
**Distal third radius T score**	-1.5± 0.7	-1.0 ± 1.0	0.130
**Femoral T score**	-1.3 ± 0.7	-1.3 ± 0.7	0.968
**Lumbar Z score**	-0.6 ± 1.2	0.1 ± 0.9	0.154
**Distal third radius Z score**	0.11 ± 0.82	0.33 ± 0.8	0.430
**Femoral Z score**	-0.11 ± 0.6	0.01 ± 0.7	0.892
**Positive imaging** (US and/or SestaMIBI)	64.4%	65%	0.279
**10-year fracture risk by FRAX** (%): Major osteoporotic Hip	- 10.1 ± 8.6 4.2 ± 6.4	- 9.2 ± 7.3 3.7 ± 5.3	- 0.641 0.667
**Prevalence of lumbar osteopenia**	22.2%	20.5%	0.136
**Prevalence of femoral osteopenia**	23.8%	20%	0.098
**Prevalence of distal third radius osteopenia**	62%	33%	**0.030**

**Note:** Data are expressed as mean ± SD when normally distributed, median and [IQR] when not normally distributed and as absolute number and percentage when categorical. *Bold points to statistically significant difference. **Data are reported for completeness, the differences in these variables were expected, being by definition the dividing variables for the 2 groups.

## Data Availability

Not applicable.

## LIST OF ABBREVIATIONS

25OHD: 25-hydroxy-vitamin D
ALP: Alkaline Phosphatase
aPHPT: Asymptomatic PHPT
bALP: Bone-specific Alkaline Phosphatase
BMD: Bone Mineral Density
Ca: Calcium
DXA: Dual X-ray Absorptiometry
eGFR: Estimated Glomerular Filtration Rate
FRAX: Fracture Risk Assessment Tool
HF: Hip Fracture
IQR: Interquartile Range
OF: Osteoporotic Fracture
OP: Osteoporosis
P: Phosphate
PHPT: Primary Hyperparathyroidism
Post-menopausal: Post-menopausal Women
PTH: Parathyroid Hormone
PTX: Parathyroidectomy
RIA: Radioimmunoassay
SD: Standard Deviation
UCa: Urinary Ca

## STANDARDS OF REPORTING

STROBE guidelines were followed.

## References

[1] Marcocci C., Cetani F. (2011). Primary hyperparathyroidism.. N. Engl. J. Med..

[2] Bilezikian J.P., Bandeira L., Khan A., Cusano N.E. (2018). Hyperparathyroidism.. Lancet.

[3] Castellano E., Attanasio R., Boriano A., Pellegrino M., Garino F., Gianotti L., Borretta G. (2017). Sex difference in the clinical presentation of primary hyperparathyroidism: Influence of menopausal status.. J. Clin. Endocrinol. Metab..

[4] Saponaro F., Cetani F., Repaci A., Pagotto U., Cipriani C., Pepe J., Minisola S., Cipri C., Vescini F., Scillitani A., Salcuni A., Palmieri S., Eller-Vainicher C., Chiodini I., Madeo B., Kara E., Castellano E., Borretta G., Gianotti L., Romanelli F., Camozzi V., Faggiano A., Corbetta S., Cianferotti L., Brandi M.L., De Feo M.L., Palermo A., Vezzoli G., Maino F., Scalese M., Marcocci C. (2018). Clinical presentation and management of patients with primary hyperparathyroidism in Italy.. J. Endocrinol. Invest..

[5] Bilezikian J.P., Khan A.A., Silverberg S.J., Fuleihan G.E.H., Marcocci C., Minisola S., Perrier N., Sitges-Serra A., Thakker R.V., Guyatt G., Mannstadt M., Potts J.T., Clarke B.L., Brandi M.L. (2020). Evaluation and management of primary hyperparathyroidism: Summary statement and guidelines from the fifth international workshop.. J. Bone Miner. Res..

[6] Coughlan T., Dockery F. (2014). Osteoporosis and fracture risk in older people.. Clin. Med..

[7] Rossini M., Adami S., Bertoldo F., Diacinti D., Gatti D., Giannini S., Giusti A., Malavolta N., Minisola S., Osella G., Pedrazzoni M., Sinigaglia L., Viapiana O., Isaia G.C. (2016). Guidelines for the diagnosis, prevention and management of osteoporosis.. Reumatismo.

[8] Kanis J.A. (2008). World Health Organization Scientific Group. Assessment of osteoporosis at the primary healthcare level. Technical Report.. WHO Collaborating Centre, University of Sheffield, UK..

[9] Marcocci C., Brandi M.L., Scillitani A., Corbetta S., Faggiano A., Gianotti L., Migliaccio S., Minisola S. (2015). Italian society of endocrinology consensus statement: Definition, evaluation and management of patients with mild primary hyperparathyroidism.. J. Endocrinol. Invest..

[10] Castellano E., Attanasio R., Boriano A., Borretta V., Tassone F., Borretta G. (2021). Diabetes and bone involvement in primary hyperparathyroidism: Literature review and our personal experience.. Front. Endocrinol..

[11] Castellano E., Attanasio R., Boriano A., Borretta G. (2019). Clinical presentation of primary hyperparathyroidism in older adults.. J. Endocr. Soc..

[12] (2002). National Kidney Foundation K/DOQI clinical practice guidelines for chronic kidney disease: Evaluation, classification, and stratification.. Am. J. Kidney Dis..

[13] Kanis J.A., Kanis J.A. (1994). Assessment of fracture risk and its application to screening for postmenopausal osteoporosis: Synopsis of a WHO report.. Osteoporos. Int..

[14] Rubin M.R., Lee K.H., McMahon D.J., Silverberg S.J. (2003). Raloxifene lowers serum calcium and markers of bone turnover in postmenopausal women with primary hyperparathyroidism.. J. Clin. Endocrinol. Metab..

[15] Nilsson S., Koehler K.F., Gustafsson J.Å. (2011). Development of subtype-selective oestrogen receptor-based therapeutics.. Nat. Rev. Drug Discov..

[16] Silverberg S.J., Clarke B.L., Peacock M., Bandeira F., Boutroy S., Cusano N.E., Dempster D., Lewiecki E.M., Liu J.M., Minisola S., Rejnmark L., Silva B.C., Walker M.D., Bilezikian J.P. (2014). Current issues in the presentation of asymptomatic primary hyperparathyroidism: Proceedings of the Fourth International Workshop.. J. Clin. Endocrinol. Metab..

[17] Silva B.C., Cusano N.E., Bilezikian J.P. (2024). Primary hyperparathyroidism.. Best Pract. Res. Clin. Endocrinol. Metab..

[18] Seib C.D., Meng T., Suh I., Cisco R.M., Lin D.T., Morris A.M., Trickey A.W., Kebebew E. (2021). Undertreatment of primary hyperparathyroidism in a privately insured US population: Decreasing utilization of parathyroidectomy despite expanding surgical guidelines.. Surgery.

[19] Nilsson M., Ståhl E., Åkesson K.E., Thier M., Nordenström E., Almquist M., Bergenfelz A. (2022). Reduced fracture incidence in patients having surgery for primary hyperparathyroidism.. Clin. Endocrinol..

[20] Seib C.D., Suh I., Meng T., Trickey A., Smith A.K., Finlayson E., Covinsky K.E., Kurella Tamura M., Kebebew E. (2021). Patient factors associated with parathyroidectomy in older adults with primary hyperparathyroidism.. JAMA Surg..

[21] Khan R., Martin J., Das G. (2021). The impact of observation versus parathyroidectomy on bone mineral density and fracture risk determined by frax tool in patients with primary hyperparathyroidism.. J. Clin. Densitom..

[22] Lundstam K., Heck A., Godang K., Mollerup C., Baranowski M., Pernow Y., Aas T., Hessman O., Rosén T., Nordenström J., Jansson S., Hellström M., Bollerslev J. (2017). Effect of surgery versus observation: Skeletal 5-year outcomes in a randomized trial of patients with primary HPT (the SIPH study).. J. Bone Miner. Res..

[23] Yeh M.W., Zhou H., Adams A.L., Ituarte P.H.G., Li N., Liu I.L.A., Haigh P.I. (2016). The relationship of parathyroidectomy and bisphosphonates with fracture risk in primary hyperparathyroidism.. Ann. Intern. Med..

[24] Ejlsmark-Svensson H., Rolighed L., Harsløf T., Rejnmark L. (2021). Risk of fractures in primary hyperparathyroidism: A systematic review and meta-analysis.. Osteoporos. Int..

[25] Seib C.D., Meng T., Suh I., Harris A.H.S., Covinsky K.E., Shoback D.M., Trickey A.W., Kebebew E., Tamura M.K. (2022). Risk of fracture among older adults with primary hyperparathyroidism receiving parathyroidectomy *vs* nonoperative management.. JAMA Intern. Med..

[26] Rao D.S., Phillips E.R., Divine G.W., Talpos G.B. (2004). Randomized controlled clinical trial of surgery versus no surgery in patients with mild asymptomatic primary hyperparathyroidism.. J. Clin. Endocrinol. Metab..

[27] Ambrogini E., Cetani F., Cianferotti L., Vignali E., Banti C., Viccica G., Oppo A., Miccoli P., Berti P., Bilezikian J.P., Pinchera A., Marcocci C. (2007). Surgery or surveillance for mild asymptomatic primary hyperparathyroidism: A prospective, randomized clinical trial.. J. Clin. Endocrinol. Metab..

[28] Bollerslev J., Jansson S., Mollerup C.L., Nordenström J., Lundgren E., Tørring O., Varhaug J.E., Baranowski M., Aanderud S., Franco C., Freyschuss B., Isaksen G.A., Ueland T., Rosen T. (2007). Medical observation, compared with parathyroidectomy, for asymptomatic primary hyperparathyroidism: A prospective, randomized trial.. J. Clin. Endocrinol. Metab..

[29] Pretorius M., Lundstam K., Heck A., Fagerland M.W., Godang K., Mollerup C., Fougner S.L., Pernow Y., Aas T., Hessman O., Rosén T., Nordenström J., Jansson S., Hellström M., Bollerslev J. (2022). Mortality and morbidity in mild primary hyperparathyroidism: Results from a 10-year prospective randomized controlled trial of parathyroidectomy versus observation.. Ann. Intern. Med..

[30] El Miedany Y. (2020). FRAX: re-adjust or re-think.. Arch. Osteoporos..

[31] (2021). Management of osteoporosis in postmenopausal women: The 2021 position statement of The North American Menopause Society.. Menopause.

[32] Khosla S., Melton J. (2002). Fracture risk in primary hyperparathyroidism.. J. Bone Miner. Res..

[33] Aspray T.J., Hill T.R. (2019). Osteoporosis and the ageing skeleton.. Subcell. Biochem..

[34] Seib C.D., Chomsky-Higgins K., Gosnell J.E., Shen W.T., Suh I., Duh Q.Y., Finlayson E. (2018). Patient frailty should be used to individualize treatment decisions in primary hyperparathyroidism.. World J. Surg..

[35] Castellano E., Attanasio R., Boriano A., Borretta G. (2018). The clinical presentation of primary hyperparathyroidism: A southern european perspective over the last 2 decades.. Endocr. Pract..

[36] Rubin M.R., Bilezikian J.P., McMahon D.J., Jacobs T., Shane E., Siris E., Udesky J., Silverberg S.J. (2008). The natural history of primary hyperparathyroidism with or without parathyroid surgery after 15 years.. J. Clin. Endocrinol. Metab..

